# Glycosylation on envelope glycoprotein of duck Tembusu virus affects virus replication *in vitro* and contributes to the neurovirulence and pathogenicity *in vivo*

**DOI:** 10.1080/21505594.2021.1974329

**Published:** 2021-09-10

**Authors:** Dejian Liu, Xuyao Xiao, Peng Zhou, Huijun Zheng, Yaqian Li, Hui Jin, Anan Jongkaewwattana, Rui Luo

**Affiliations:** a State Key Laboratory of Agricultural Microbiology, College of Veterinary Medicine, Huazhong Agricultural University, Wuhan, Hubei, China; b Key Laboratory of Preventive Veterinary Medicine in Hubei Province, the Cooperative Innovation Center for Sustainable Pig Production, Wuhan, Hubei, China; c Virology and Cell Technology Research Team, National Center for Genetic Engineering and Biotechnology (Biotec), National Science and Technology Development Agency (Nstda), Klong Nueng, Pathum ThaniThailand

**Keywords:** Duck Tembusu virus, envelope protein, glycosylation, replication, pathogenicity

## Abstract

Duck Tembusu virus (DTMUV), an emergent flavivirus, causes domestic waterfowls to suffer from severe egg-drop syndrome and fatal encephalitis, greatly threatens duck production globally. Like other mosquito-borne flaviviruses, the envelope (E) protein of all DTMUV strains was N-glycosylated at the amino acid position 154. Thus far, the biological roles of DTMUV E glycosylation have remained largely unexplored. Herein, we demonstrated the key roles of E glycosylation in the replication and pathogenicity of DTMUV in ducks by characterizing the reverse-genetics-derived DTMUV wild-type MC strain and MC bearing mutations (N154Q and N154I) that abolish the E glycosylation. Our data showed that the disruption of E glycosylation could substantially impair virus attachment, entry, and infectivity in DEFs and C6/36 cells. Notably, ducks inoculated intracerebrally with the wild-type virus exhibited severe disease onset. In contrast, those inoculated with mutant viruses were mildly affected as manifested by minimal weight loss, no mortality, lower viral loads in the various tissues, and reduced brain lesions. Attenuated phenotypes of the mutant viruses might be partly associated with lower inflammatory cytokines expression in the brains of infected ducks. Our study offers the first evidence that E glycosylation is vital for DTMUV replication, pathogenicity, and neurovirulence *in vivo*.

## Introduction

Tembusu virus (TMUV), a group member of the Ntaya virus within the genus *Flavivirus* in the family *Flaviviridae*, was initially separated in 1955 from *Culex tritaeniorhynchus* mosquitoes in Malaysia. In 2010, TMUV caused the sudden outbreak of duck egg drop syndrome (DEDS) in southeast China and the disease rapidly spread throughout the country [1]. Shortly thereafter, outbreaks of duck TMUV (DTMUV) infection among domestic ducks were reported in Malaysia [2] and Thailand [3]. Besides the egg drop syndrome, the DTMUV-infected ducks also display retarded growth, acute anorexia, and neurologic symptoms. DTMUV can infect all duck species with nearly 100% morbidity and 10% to 30% mortality, causing massive economic losses to the duck industry [1,4–6]. TMUV has been shown to exhibit a broad natural host range, including mosquitoes and various avian species, such as chickens, ducks, geese, pigeons, and sparrows [6]. Like most flaviviruses, the mosquito was considered a crucial component in the transmission cycle of DTMUV [7]. Of note, DTMUV causes diseases in egg-laying ducks that can spread across northern China, even in the winter when mosquito activity is relatively low, suggesting that non-vector transmission routes might be involved in TMUV spread [1,8]. Recently, a combined epidemiological and experimental investigations have revealed that DTMUV can infect ducks through direct contact and aerosol spread [8].

DTMUV possesses a positive, single-stranded RNA genome (about 11 kb), encoding a single polyprotein. Viral and host proteases cut this polyprotein into three structural proteins, including the capsid (C), pre-membrane (prM), and envelope (E) proteins, as well as seven nonstructural (NS) proteins (NS1, NS2A, NS2B, NS3, NS4A, NS4B, and NS5) [9]. The processes of viral attachment, membrane fusion, and virion assembly are mediated by the structural proteins; the NS proteins are associated with viral replication as well as the modulation of innate and adaptive immune responses from host [10–14]. The flavivirus replication cycle begins when the virus binds to its host cells via the interaction between the viral E protein and receptors on the cell surface [15]. Soon after viral attachment, the virion is internalized via receptor-mediated endocytosis [16]. Upon exposure to the late endosome’s acidic environment, conformational changes in E protein occur, resulting in viral and cellular membrane fusion [16]. After membrane fusion, the viral RNA is uncoated in the cytoplasm and translated by the host’s machinery [17]. Viral genome replication occurs within compartments derived from membrane invagination of the endoplasmic reticulum (ER) so-called vesicle packets (VPs). The newly synthesized genome is associated with the capsid to form the nucleocapsid (NC) and then assembles with prM and E proteins on the ER’s surface juxtaposed to the VPs. This immature virion buds into the ER lumen and eventually pass through the secretory pathway [18]. Proteolysis of prM by host furin during Golgi transit leads to the homodimerization of E, resulting in infectious virion maturation [19]. Finally, infectious viral particles are released from the host cell via exocytosis [17].

Flaviviruses contain several asparagine (N)-glycosylation sites (N-X-S/T) located in the NS1, prM, and E proteins [20–22]. The NS1 proteins of most vector-borne flaviviruses, such as Zika virus (ZIKV), dengue virus (DENV), and Japanese encephalitis virus (JEV), have two N-glycosylation sites at N130 and N207 [20,23,24]. Several members possess a third putative glycosylation site at N175, including West Nile virus (WNV), St. Louis encephalitis virus (SLEV), and DTMUV [20]. Glycosylation at N130 in DENV NS1 was found to be critical for viral replication and stabilization of the secreted hexamer, whereas the N-glycosylation at residue 207 promotes the NS1 secretion and stability [25]. Ablation of the three N-glycosylation sites in WNV NS1 protein causes the accumulation of NS1 in the endoplasmic reticulum and significantly impairs viral replication [26].

Unlike NS1, the prM protein contains a single putative N-glycosylation site located at different positions among various flaviviruses. For JEV, the glycosylation site at N15 in the prM is highly conserved among different strains. Removal of the N15 glycosylation blocks JEV growth at the stage of virus release and results in a dramatic decrease in virulence in mice [21]. Recently, Gwon et al. reported that N69 glycosylation of ZIKV prM is essential for effective secretion of virions, and involves in viral protein tracking and virion assembly [27].

The flavivirus E protein is a major surface glycoprotein closely associated with important stages of viral survival and proliferation, especially viral attachment, entry, and assembly [9]. Generally, the E protein of vector-borne flaviviruses has up to two distinct N-glycosylation sites, depending on the virus [22]. The N153/154 of E proteins is usually decorated by N-linked glycans among most flaviviruses, including Zika virus (ZIKV), dengue virus (DENV), West Nile virus (WNV), and Japanese encephalitis virus (JEV), while N67 is unique to DENV [22,28]. However, the glycosylation pattern on the E protein is not completely conserved among all flaviviruses and even among different strains of the same virus [22]. For example, all Asian lineage ZIKV strains contain the N154 glycosylation site of the E protein, whereas E glycosylation is ablated in some African lineage ZIKV, such as MR766 [29–31]. Similarly, many WNV strains display glycosylation at N154, while others contain no glycosylation site due to mutations at N154 or S/T156 [32,33]. All TMUV isolates exhibit N154 glycosylation of the E proteins except for TMUV prototype strain MM1775, where the mutation S156P of E protein disrupt the N-linked glycosylation at N154 [34].

It has been shown that the N-glycosylation within an N-X-S/T sequon of the flavivirus E protein strongly impacts viral replication efficiency, neurovirulence, and pathogenesis [22]. For instance, the absence of the N-glycosylation site of ZIKV E protein has little effect on replication in mammalian cell culture but significantly augmented growth in mosquito cells [35]. Moreover, the E glycosylation is critical for viral virulence in mice, but, interestingly, not associated with the neurovirulence [35]. Beasley et al. demonstrated that the N-glycosylation on the WNV E protein is related to increased neuroinvasion and virulence in mice, suggesting that E glycosylation promotes trafficking of the virus across the blood–brain barrier [36]. On the contrary, glycosylated and non-glycosylated WNV exhibit similar virulence in avian, such as American crows and house sparrows [33]. More importantly, it has been recently reported that the mutation S156P of DTMUV E protein could disrupt the N-linked glycosylation at N154, resulting in the abrogation of vector-free transmission of DTMUV among ducks. Although it has been indicated that the E glycosylation affect viral pathogenesis, its in vivo biological function remains largely undetermined. In this study, we developed a reverse genetic platform for DTMUV to investigate how viral replication in cell cultures was impacted by E glycosylation. Furthermore, its role in pathogenicity, especially neurovirulence, in infected ducks was also explored.

## Materials and methods

### Ethics statement

The animal experiments were reviewed and approved by the Animal Ethics Committee of Huazhong Agricultural University (HZAUDU-2018-005).

### Cells, viruses, and antibodies

Duck embryo fibroblast (DEF, #CCL-141) and Aedes albopictus C6/36 (#CRL-1660) cells were received from the American Type Culture Collection (ATCC). DEF cells were cultured in the Minimum Essential Medium (MEM, Gibco) supplemented with 10% fetal bovine serum (FBS, Gibco) at 37°C. RPMI 1640 medium (Gibco) supplemented with 10% FBS was used to culture C6/36 cells at 28°C. Duck glial cells were isolated from new-born duck brains and cultured in Dulbecco’s Modified Eagle Medium (DMEM, Gibco) containing 10% FBS at 37°C. DTMUV strain MC (GenBank number: KX452096) was stored in our lab and propagated in DEFs in MEM medium with 2% FBS. The monoclonal antibody, 3F12, against DTMUV E protein was generated previously in our lab.

### Construction of DTMUV full-length cDNA clone

**Figure 1.**
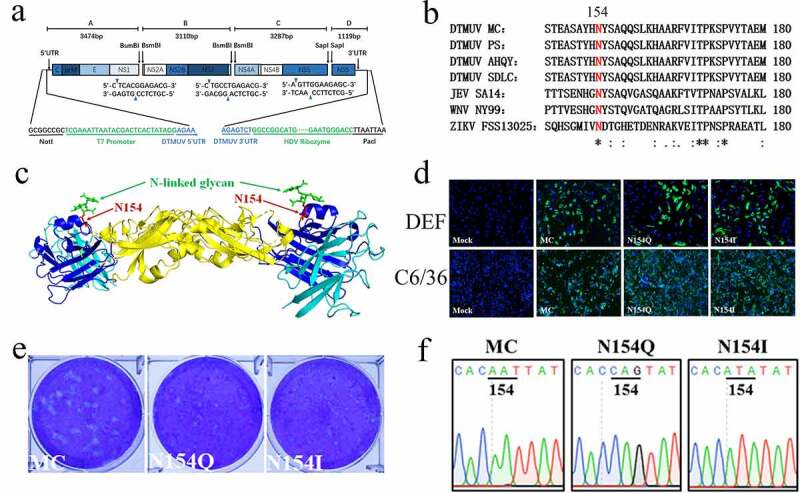
(a) Schematic diagram of DTMUV MC infectious clone. The DTMUV MC genome was split into 4 segments by the restriction endonucleases and cloned into pJET1.2/blunt vectors. A T7 promoter and a hepatitis delta virus (HDV) ribozyme were placed to the immediate 5ʹ and 3ʹ ends of the viral genome. (b) Alignment of amino acid sequence containing the N-glycosylation site at N154 on flavivirus E proteins. The E glycosylation regions from DTMUV strains MC (GenBank: KX452096.1), PS (GenBank: KT876991.1), AHQY (GenBank: KJ740748.1), and SDLC (GenBank: KJ740747.1), JEV strain SA14 (GenBank: D90194.1), WNV strain NY99 (GenBank: DQ211652.1) and ZIKV strain FSS13025 (GenBank: KU955593.1) were compared. The N-glycosylation site at N154 was conserved and highlighted in red. (c) Structure of DTMUV E dimer. The structure was modeled by SWISS-MODEL online using the West Nile virus E protein crystal structure (PDB ID: 3IYW). Domain I, II, and III on E were shown in blue, yellow, and cyan, respectively. The N154 residues and glycans on each monomer are displayed in red and green, respectively. (d) IFA of the E protein expression in cells infected with MC and N154Q/I viruses. DEFs and C6/36 cells were inoculated with MC and N154Q/I at 0.01 MOI. At 36 hpi, IFA was employed to determine E protein expression in DEFs and C6/36 cells using the mouse anti-E mAb (3F12). Nuclei were stained with DAPI. Green and blue represent E protein and nuclei. (e) Plaque morphologies of MC and N154Q/I. (f) Chromatogram analysis of the E glycosylation regions of MC and N154Q/I

We used a quadripartite unidirectional molecular clone strategy to develop a full-length infectious cDNA clone of the DTMUV MC strain, similar to the strategy previously reported for ZIKV [37]. As illustrated in Figure 1(a), the full-length viral genome was split into four consecutive fragments which can be systematically ligated by two BsmBI restriction sites at nucleotide positions 3474, 6584 and one SapI restriction site at nucleotide position 9871. A T7 transcription promoter was added to the immediate 5ʹ end of the viral genome, while a hepatitis delta virus (HDV) ribozyme sequence was also added to the 3ʹ end. Four cDNA fragments (A-D, Figure 1(a)) were synthesized and cloned into the pJET1.2/blunt cloning vector (Thermo Scientific) based on the genome sequence of DTMUV strain MC (GenBank number: KX452096) and amplified in *Escherichia coli* strain DH10B. All plasmids containing DTMUV fragments were verified by Sanger sequencing. The DTMUV A-D inserts were digested with restriction endonucleases indicated in Figure 1(a), isolated through agarose gels and extracted by a QIAquick gel extraction kit (Qiagen). These prepared fragments were mixed and ligated overnight using T4 DNA ligase (Thermo Scientific) at 4°C. After extraction with the solution of phenol in chloroform, full-length DTMUV RNA was generated by *in vitro* transcription using mMESSAGE mMACHINE T7 Transcription Kit (Ambion) following the manufacturer’s instructions. RNA transcripts were transfected into DEFs (8.0 × 10^6^ cells/ml) in PBS, and triple electrical pulses (450 V at 50 µF) were performed using a Gene Pulser X cell electroporation system (Bio-Rad). The electrical pulsed cells cells were transferred into a flask and cultured for 48 h. Rescued viruses were harvested and further purified by plaque assay.

To remove the N-glycosylation of DTMUV E protein, E N154Q (A^1415^AT^1417^ to C^1415^AG^1417^) and E N154I (AA^1416^T^1417^ to AT^1416^A^1417^) mutations were introduced into the DTMUV MC infectious clone. Mutations were introduced to the DTMUV fragment A with the primers listed in Table S1 in Supplemental Material. Subsequently, the mutated fragments were digested, ligated with B-D fragments, *in vitro* transcribed, and electroporated to DEFs. Following harvest and plaque purification of rescued virus, the sequences of all recombinant viruses were confirmed by genome sequencing.

### Virus genome sequencing

Viral RNA was extracted using the QIAamp Viral RNA Mini Kit, and the viral genome RNA was reverse transcribed into cDNA by SuperScript III transcriptase (Invitrogen, USA) using random primers. The primers (DTMUV-5′GSP and DTMUV-3′GSP, Table S1) were employed to confirm the 5′ and 3′ end sequences of N154Q/I mutants using the SMARTer RACE 5′/3′ Kit (Clontech) following manufacturer’s instructions. The whole genome of N154Q/I was sequenced with primers listed in Table S1. Sequence assembly was performed by Seqman (DNAstar).

### Indirect immunofluorescence assay

DEF and C6/36 cells were cultured on sterilized coverslips before being infected with DTMUV. After 36 h post-infection, cells were fixed in 4% formaldehyde at room temperature and subsequently permeabilized with 0.1% Triton X-100 (Sigma). After blocking, cells were treated with mouse anti-DTMUV E antibodies, clone 3F12, followed by incubation with Alexa 488 goat anti-mouse IgG (Thermo Fisher Scientific). For nucleus staining, cells were incubated with DAPI (Invitrogen). Fluorescence was collected by a fluorescence microscope (Olympus IX73, Japan).

### Plaque assay

DEFs were cultured in 6-well plates and inoculated with wild-type MC and N154Q/I mutants, respectively. Following 10-fold serial dilution, viral samples were inoculated into the DEFs cultured in the plates; each infection was repeated 3 times. After 1 h incubation, samples were eliminated, and DEFs were covered by 3 mL of a 1:1 mixture of 1.6% agarose, and 2x MEM (Gibco) containing 2% FBS and 1% penicillin/streptomycin. After 60 h incubation, DEFs were fixed with 4% formaldehyde and stained with 1% crystal violet for 1 h. Plaques were counted to determine the viral titers.

### Viral growth kinetics

DEF and C6/36 cells were inoculated with 0.01 MOI MC, and N154Q/I mutants; each infection was repeated 3 times. After 1 h incubation, cells were washed with cold PBS and grown in a fresh medium. The plates were incubated for 72 h, and the cell cultures were collected every 12 hours for virus titration. Virus titers were measured by TCID_50_ endpoint dilution assay and calculated by Reed and Muench’s method.

### Western blotting and glycosidase treatment

Cells were infected with MC and N154Q/I mutants and collected by RIPA lysis buffer, followed by centrifugation at 12,000 xg for 15 min. Aliquots of the cell lysates were treated with PNGase F (New England BioLabs). Proteins extracted from the lysates were separated by 12% SDS-PAGE, and transferred to a polyvinylidene difluoride (PVDF) membrane. Blots were incubated with PBST containing 10% skim milk, followed by incubation with mouse anti-DTMUV E antibodies. After washing with PBST buffer, the membranes were incubated with HRP-conjugated goat anti-mouse IgG (ABclonal). Finally, luminescence was produced using the Clarity^TM^ Western ECL Substrate (Bio-Rad).

### Quantification of extracellular and intracellular infectious virions

Quantification of infectious virions was carried out following the previous protocol [35]. Briefly, cell supernatants were then harvested from the infected cells at indicated time points and centrifuged to eliminate cell debris before storage at −80°C. Infected cells were washed with cold PBS to eliminate unbound virions, while virions binding to the cell surface was eliminated by stringent washes with the cold alkaline-high-salt solution. After washing with cold PBS, infected cells were digested using 0.25% Trypsin and suspended in MEM supplement with 2% FBS. After centrifugation, cell pellets were resuspended in 0.25 mL MEM. The suspended cells (100 μL) were used for intracellular viral RNA extraction. The remaining cells were lysed by three freeze-thaw cycles and subjected to test the intracellular infectivity by plaque assay.

### Duck experiments

To compare the pathogenicity of N154Q/I mutants with MC in ducks, three-day-old Cherry Valley ducks were intracerebrally (i.c.) inoculated with 10^5^ TCID_50_ of MC and N154Q/I; 10 ducks per group. A group of 10 control ducks was inoculated with equal volumes of PBS. Clinical symptoms, weight changes, and survival rates in each group were monitored and recorded daily after inoculation.

To compare the viral proliferation and neurovirulence of N154Q/I mutants with the MC in ducks, 10^5^ TCID_50_ of each virus was inoculated i.c. into 15 ducks. Three ducks from each group were euthanized humanely at 2, 4, 6 days post-inoculation (dpi). Sera and tissue samples from the brain, spleen, and liver were collected for viral load detection using SYBR Green-based qRT-PCR assays. The expression of cytokines and chemokines in these brain tissues were also quantified by qRT-PCR. Brains collected from the ducks on day 6 post-inoculation were sectioned along the midline, with one-half used for qRT-PCR and the other half used for histopathological analysis.

### Real‑time qRT‑PCR

To determine the viral load, tissues were weighed and homogenized in DMEM (0.2 g tissue per 1 mL of DMEM). Homogenized extracts, sera, or cell cultures were harvested and clarified by centrifugation, and the supernatants were collected for RNA extraction with the Trizol reagent (Invitrogen). RNA was converted into single-stranded cDNA with the random primers using the Transcriptor First Strand cDNA Synthesis Kit (Roche). DTMUV RNA copies were determined using a primer set (DTMUV-qF, DTMUV-qR; Table S1). The DTMUV E gene was cloned into the pCAGGS-HA vector to construct a standard plasmid. Applied Biosystems 7500 Fast Real-time PCR System (Life Technologies, USA) was employed to perform qRT-PCR with FastStart Universal SYBR Green Master (Roche). Cycling conditions were used as follows: 95°C for 10 min, followed by 40 cycles of 95°C for 15 s, and 60°C for 45 s. In each assay, tenfold serial dilutions of the recombinant plasmid (pCAGGS-HA-E) was used to generate a linear standard curve and distilled water as negative control. All samples were tested in three independent assays. The quantity of DTMUV RNA was determined by normalizing with pCAGGS-HA-E. The number of viral RNA copies in the sera and tissue samples were calculated as viral RNA copies per milliliter of serum or per gram of tissues.

To quantify the expression of proinflammatory cytokines in the brain, samples were briefly homogenized in DMEM (0.2 g tissue/1 mL DMEM), and 0.2 mL of supernatant was used for RNA extraction using the Trizol reagent (Invitrogen), followed by reverse-transcription into cDNA with oligo(dT) primers. The abundances of proinflammatory cytokines were quantified by SYBR-based qPCR (Roche). Primers used in real-time PCR were listed in Table S1. The abundances of individual transcripts were tested in three independent assays and normalized to duck GAPDH (AY436595.1) mRNA level. The amplification conditions of cDNA were: 95°C for 10 min, followed by 40 cycles of 95°C for 15 s, and 60°C for 60 s.

### Histology analysis

Sections of the brain obtained from the different groups were fixed in 10% formalin for 48 h. Following dehydration and embedding, tissue samples were sectioned. After mounting the tissue sections on the glass slides, they were stained with hematoxylin and eosin (H&E). Finally, the slides were examined with conventional microscopy.

### Statistical analysis

Data reported in the figures were analyzed using Prism 6 (GraphPad Software). An unpaired Student’s *t*-test was applied to determine the *p*-value. *p*-values <0.05 were regarded as statistically significant and *p*-values <0.01 were highly significant.

## Results

### Development of a DTMUV reverse genetics system and recovery of DTMUV mutants lacking E glycosylation

Recently, the reverse genetics system for ZIKV has been successfully developed using a unidirectional assembly of a quadripartite genome [37], which is an approach widely utilized to develop infectious clones of positive-sense RNA viruses including coronaviruses [38,39]. Here, we adapted this strategy to generate the infectious clone of DTMUV strain MC. As shown in Figure 1(a), the complete viral genome was divided into four consecutive segments (A-D) that could be unidirectionally tethered by two BsmBI restriction sites (nucleotide positions 3474 and 6584) and one SapI restriction site (nucleotide position 9871). Both BsmBI and SapI are type IIS restriction endonucleases that cleave at short specific distances from their recognition site and leaves the non-palindromic sequence overhangs. Thus, these four segments can be ligated into the full-length DTMUC genomic cDNA with high efficiency. A T7 RNA polymerase promoter sequence was introduced into the extreme 5ʹ terminus of the fragment A, which was used for *in vitro* transcription from the ligated full-length cDNA clones. In addition, a hepatitis delta virus (HDV) ribozyme was positioned following the last DTMUV nucleotide in the fragment D to create an authentic DTMUV genomic 3ʹ-end (Figure 1(a)).

Multiple sequence alignment shows that a unique conserved N-linked glycosylation site (N-X-T/S) at residue N154 was universally found on the surface of domain I of the E protein from DTMUV isolates (Figure 1(b,c)). To specifically investigate the role of DTMUV E glycosylation, we generated N154Q (A^1415^AT^1417^ to C^1415^AG^1417^) and N154I (AA^1416^T^1417^ to AT^1416^A^1417^) mutations in the fragment A by site-directed mutagenesis to abolish the E glycosylation. Following *in vitro* ligation and transcription, these full-length transcripts were electroporated into DEFs and apparent cytopathic effects were observed 72 h post-transfection in transfected cells. Results from indirect immunofluorescence assays (IFA) using an E-specific monoclonal antibody in both DEFs and C6/36 cells infected with the rescued viruses confirm the success of virus recovery (Figure 1(d)). It is, however, important to note that N154Q/I exhibited reduced plaque size compared to MC (Figure 1(e)). After three rounds of plaque purification, the genomic RNA of rescued N154Q/I was subject to nucleotide sequencing. Sequencing the complete genome of N154Q/I proved that no reversion or unwanted mutations occurred in engineered mutation (Figure 1(f)). Notably, after at least three successive passages of the rescued viruses on DEFs, the genomes of engineered N154Q/I viruses were stable, suggesting that E-glycosylation-knockout mutants of DTMUV were successfully constructed.

### In vitro characterization of E-glycosylation knockout DTMUVs

**Figure 2.**
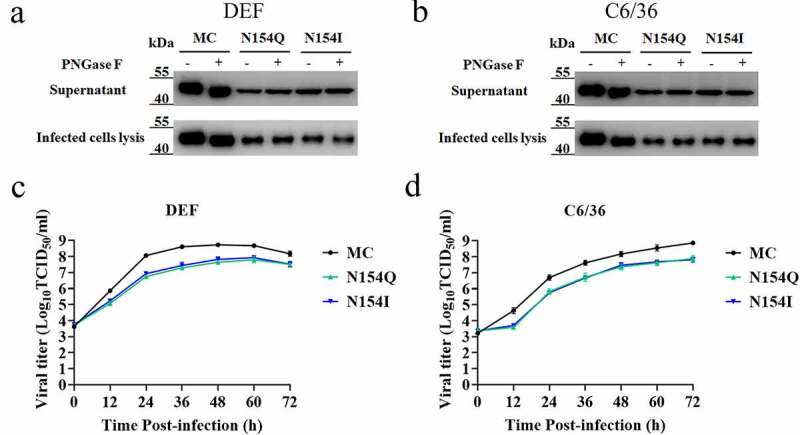
(a, b) Endoglycosidase analyses of E protein. E protein in supernatants and cell lysates was collected from MC and N154Q/I–infected DEFs (a) and C6/36 cells (b) and digested by PNGase F for 1 h at 37°C prior to Western blot analysis using the mouse anti-E mAb (3F12). Size differences of the E protein are exhibited according to their levels of glycosylation. (c, d) Growth curves of MC and N154Q/I viruses in DEFs (c) and C6/36 (d) cells. DEFs and C3/36 cells were inoculated with MC and N154Q/I (MOI = 0.01). Virus titers were determined at different timings by TCID_50_ on DEFs. Mean ± SDs from three independent experiments are displayed. ** *p* < 0.01 (unpaired Student’s *t*-test)

To assess the wild-type as well as the corresponding mutant viruses without the glycosylation on E protein, the infected supernatants and cell lysates were incubated with peptide N-glycosidase F (PNGase F) to remove N-linked oligosaccharides from the E protein. As depicted in Figure 2(a,b), western blot analysis demonstrated that the addition of PNGase F markedly enhanced the migration of E protein of the MC in both DEFs and C6/36 cells, whereas the mobility of N154Q/I E protein was not affected by PNGase F treatment (Figure 2(a,b)). These data suggested that the N-glycosylation of DTMUV E protein was absent in N154Q/I mutants.

To determine whether the E protein glycosylation affects DTMUV replication in cell cultures, we infected DEFs and C6/36 cells with the wild-type virus and the E glycosylation mutants and compared viral replication kinetics. We found that at all time points after infection N154Q/I mutants yielded substantially lower viral titers than MC on both cell types (Figure 2(c,d)). Specifically, the viral titers of N154Q /I were about 20-fold reduced in DEFs at 36 h post-infection (hpi) compared to MC (Figure 2(c)), while N154Q/I showed a 10-fold reduction in viral titers in C6/36 cells at 60 hpi (Figure 2(d)). These results collectively indicated that E protein’s glycosylation is critical for DTMUV replication in both DEFs and C6/36 cells.

### Disruption of E glycosylation impairs the attachment and entry of DTMUV as well as the infectivity of progeny in DEFs and C6/36 cells

**Figure 3.**
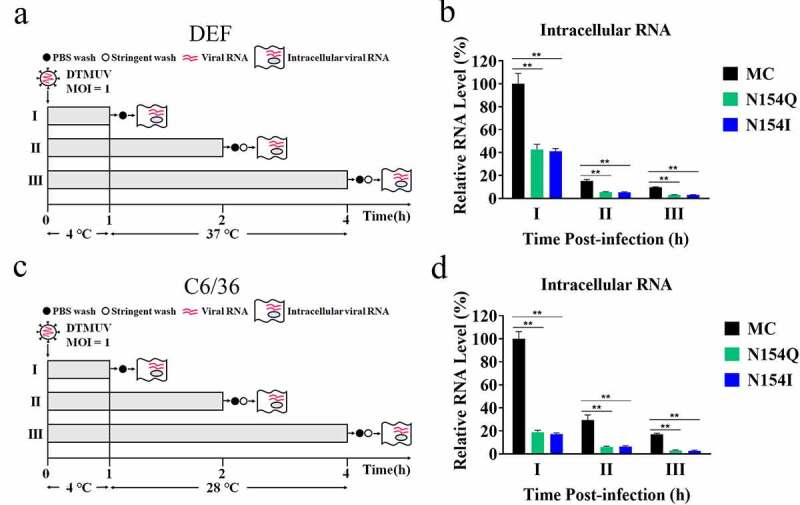
Impacts of the N154Q/I mutation on virus attachment and entry in DEFs and C6/36 cells. (a, c) Experimental design to investigate the viral attachment and entry in DEFs (a) and C6/36 cells (c). (b, d) Intracellular viral RNA isolated from virus-infected DEFs (b) and C6/36 cells (d) was quantified at indicated time points by qRT-PCR. Mean ± SDs from three independent experiments are displayed. ** *p* < 0.01 (unpaired student’s *t*-test)

To further investigate at which steps of the DTMUV replication were affected by the lack of E glycosylation, we sought to examine several important processes related to viral replication using the mutants and wild-type virus, respectively. To explore the viral attachment and entry, we inoculated DEFs or C6/36 cells with equal amounts of infectious MC, N154Q, or N154I virus at 4°C, enabling them to attach to the cell surface but not to internalize (Figure 3(a,c)). After 1 h incubation, cells from group I were washed to remove the unbound virus, and the viruses binding to the cell surface were measured by qRT-PCR. As shown in Figures 3b and 3d, compared to the MC, the amount of the N154Q/I bound to the cells was reduced by approximately 60% and 80% in DEFs and C6/36 cells, respectively. To assess the viral entry, groups II and III were further incubated at 37°C in DEFs (Figure 3(a)) or 28°C in C6/36 cells (Figure 3(c)). Infected DEFs and C6/36 cells were thoroughly washed at 2 or 4 h post-inoculation (hpi) with a highly basic and salty solution to eliminate cell membrane-associated viruses, and the intracellular viral RNA was analyzed by qRT-PCR. Consistent with the attachment assay, the amount of the N154Q/I viral RNA was lower with statistical significance than that of MC viral RNA at 2 and 4 hpi (Figure 3(b,d)), indicating that N154Q/I mutation impaired virus attachment and/or entry in DEFs and C6/36 cells.

**Figure 4.**
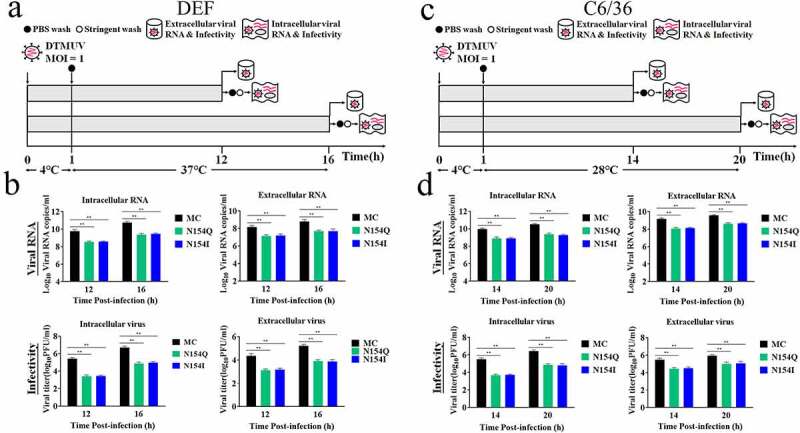
Impacts of the N154Q/I mutation on the infectivity of progeny virus in DEFs and C6/36 cells. (a, c) Experimental design to explore the infectivity of progeny virus in DEFs (a) and C6/36 cells (c). (b, d) Viral RNA and infectious virus in infected DEFs (b) and C6/36 cells (d) were determined by qRT-PCR and plaque assay. Mean ± SDs from three independent experiments are displayed. ** *p* < 0.01 (unpaired Student’s *t*-test)

To determine the role of the E glycosylation on the infectivity of progeny virus, we infected DEFs and C6/36 cells with MC, N154Q or N154I virus at 37°C or 28°C for 1 h, after which unattached viruses were removed by three washes with PBS (Figure 4(a,c)). After incubating the infected DEFs at 37°C for 12 and 16 h or C6/36 cells at 28°C for 14 and 20 h, the intracellular and extracellular viral RNAs and infectious viruses were measured by qRT-PCR and plaque assay. As shown in Figure 4(b,d), the N154Q/I virus yielded less viral RNA and infectious virions than the MC virus in both DEFs and C6/36 cells. Taken collectively, these results indicate that the glycosylation of E protein significantly affects the attachment and entry DTMUV as well as the infectivity of progeny virus in DEFs and C6/36 cells.

### E glycosylation mutants reduce inflammatory responses in DTMUV-infected duck glial cells

**Figure 5.**
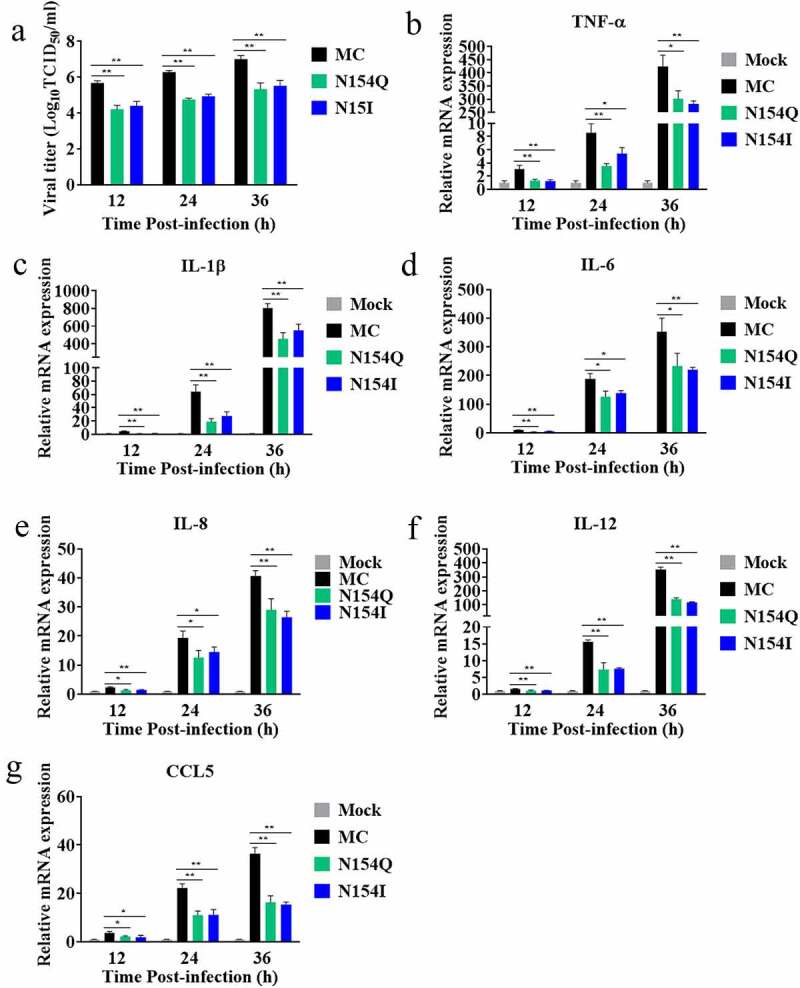
Comparison of MC and N154Q/I viruses-induced proinflammatory cytokines expression in duck glial cells. (a) Duck glial cells were isolated from one-day-old cherry valley ducks infected with MC and N154Q/I viruses. At the different timings, viral titers were measured by TCID_50_. (b-g) The relative intracellular mRNA levels of proinflammatory cytokines and chemokines, including TNF-α (b), IL-1β (c), IL-6 (d), IL-8 (e), IL-12 (f), and CCL5 (g), were measured by real-time RT-PCR using duck GAPDH for normalization. Mean ± SDs from three independent experiments are displayed. *p < 0.05 and ** *p* < 0.01 (unpaired student’s t-test)

Given that DTMUV has been shown to penetrate duck’s central nervous system and induces the production of proinflammatory cytokines in the brain [40,41], we asked whether the wild-type and mutant DTMUV could infect duck primary glial cells and differently induce inflammatory responses. To this end, we isolated glial cells from the brains of one-day-old ducks that were inoculated with wild-type or E-glycosylation-mutant viruses. Viral titer in cell cultures and the intracellular mRNA abundance of different inflammatory cytokines were measured at 12, 24, and 36 hpi, respectively. Compared to the MC virus, N154Q/I mutants produced less infectious virus (Figure 5(a)) and induced lower levels of proinflammatory cytokines (Figure 5(b,g)), including TNF-α, IL-1β, IL-6, IL-8, IL-12, and CCL5. These results indicated that the glycosylation of E protein might contribute to the DTMUV-induced neuroinflammation.

### DTMUVs lacking E glycosylation are attenuated in vivo

**Figure 6.**
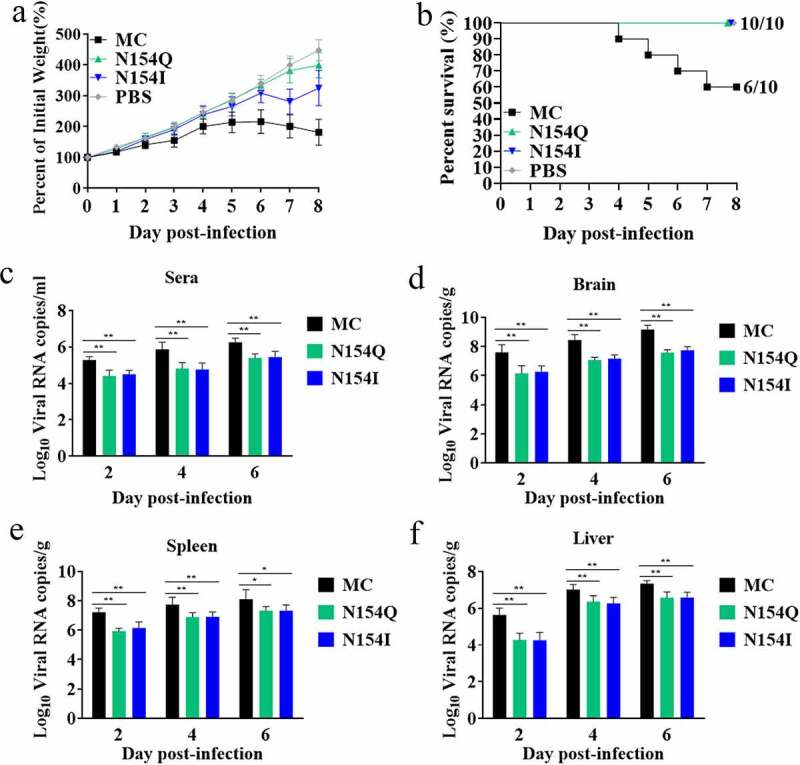
Comparison of the pathogenicity between MC and N154/I viruses in ducks. (a) Duck weight loss after inoculation of MC and N154/I viruses. (b) Mortality of MC and N154/I viruses infected-ducks. (c-f) Viral RNA copies in the serum (c), brain (d), spleen (e), and liver (f) of MC and N154/I viruses-infected ducks at 2, 4, and 6 dpi. Viral RNA copies were measured by qRT-PCR. Data are exhibited as mean ± SEMs from three independent experiments. *p < 0.05 and ** *p* < 0.01 (unpaired student’s t-test)

**Figure 7.**
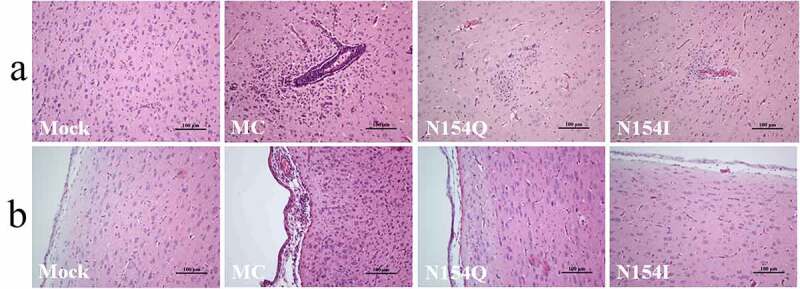
Histopathological examination of brains from MC and N154/I viruses-infected ducks. H&E staining of the cerebrum (a) and meninges (b) of ducks infected with MC and N154/I viruses. Scale bars are shown in each picture

**Figure 8.**
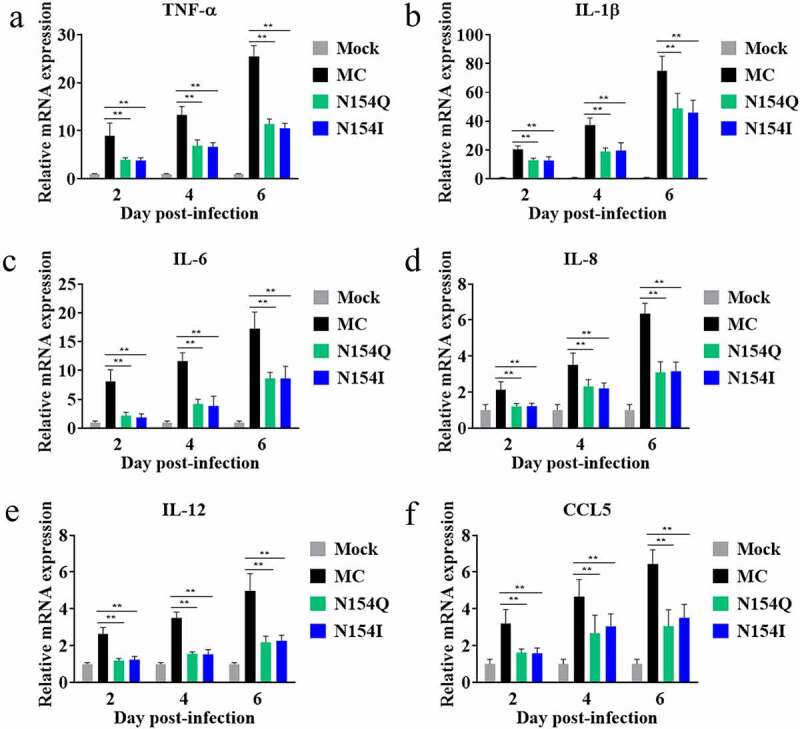
Comparison of proinflammatory cytokines expression in brains of MC and N154Q/I viruses-infected ducks. The relative mRNA levels of TNF-α (a), IL-1β (b), IL-6 (c), IL-8 (d), IL-12 (e), and CCL5 (f) in brains were measured by real-time RT-PCR using duck GAPDH for normalization. Data are exhibited as mean ± SEMs from three independent experiments. ** *p* < 0.01 (unpaired student’s t-test)

To determine the function of E protein glycosylation on DTMUV pathogenicity *in vivo*, we inoculated three-day-old ducks intracerebrally with the MC, N154Q, or N154I viruses. The MC-infected ducks exhibited depression, and paralysis starting at 3 dpi with significant weight loss observed at 6 dpi thereafter (Figure 6(a)). In contrast, the N154Q/I–infected ducks exhibited mild clinical symptoms with a slight weight loss (Figure 6(a)). Of note, while we observed no mortality in the N154Q/I–infected ducks, 40% of MC-infected ducks succumbed to the infection as early as 4 dpi (Figure 6(b)). To further investigate the effect of the E protein glycosylation on DTMUV replication *in vivo*, we inoculated three-day-old ducks with MC or N154Q/I viruses, and three ducks from each group were euthanized at 2, 4, and 6 dpi to examine the amount of virus in sera and tissues from various organs. As depicted in Figure 6(c,f), the viral load in the sera, brain, spleen, and liver obtained from N154Q/I–infected ducks were significantly lower than those from MC-infected ducks at all time points. To assess the effect of E protein glycosylation on neurovirulence in ducks, we examined the brain lesions in the MC and N154Q/I–infected ducks collected at 6 dpi. Histopathological examination of brain sections showed clear differences between N154Q/I- and MC-infected groups. Of note, MC-infected ducks exhibited perivascular lymphocytic cuffing in the brains (Figure 7(a)) and diffuse lymphocytic infiltrates in the meninges (Figure 7(b)). In contrast, minimal meningeal and perivascular inflammation was observed in N154Q/I–infected ducks (Figure 7(a,b)). We next sought to determine whether E glycosylation also affects neuroinflammatory responses in infected ducks. To this end, we quantified the expression levels of inflammatory cytokines and chemokines in the cerebral cortex of the MC and N154Q/I–infected ducks. As shown in Figure 8(a,f), N154Q/I–infected tissues exhibited substantially lower level of inflammatory cytokines and chemokines (TNF-α, IL-1β, IL-6, IL-8, IL-12, and CCL5) than those infected with MC. Collectively, these results demonstrated that the absence of the DTMUV E protein glycosylation leads to attenuated virus replication, neuroinflammation, and brain injury in ducks.

## Discussion

N-Glycans on flavivirus glycoproteins have been shown to significantly affect the viral infection and pathogenesis. With the exception of DENV E, most flaviviruses, including DTMUV, contain a single conserved N-linked glycosylation sites (N-X-S/T) at N153/154 in their E glycoproteins [22]. It has been reported previously that a specific mutation at the serine residue at position 156 to the proline could severely affect the N-linked glycosylation at N154. The mutation at S156 was found to be vital for tissue tropism and transmission in ducks [34]. In line with the previous work, we herein demonstrated, using both *in vitro* and *in vivo* assays, the crucial function of DTMUV E glycosylation at N154 in viral replication, pathogenesis, and neuroinvasiveness.

We first evaluated the viral replication efficiencies of the wild-type and mutant DTMUVs with E glycosylation knockout in cell lines from duck and mosquito. E glycosylation mutation was found to impair viral replication significantly in DEF, C6/36, and duck glial cells (Figure 2(c,d), 5(a)). Furthermore, DTMUV E glycosylation was shown to be critical for viral attachment, entry, and infectivity of progeny virus in both DEF and C6/36 cells (Figures 3 and 4). In congruence with the *in vitro* results, disruption of the E glycosylation resulted in the dramatically attenuated phenotypes of DTMUV in new-born ducks as evidenced by marginal weight loss, no mortality, reduced viremia, and decreased viral loads in various tissues (Figure 6). Despite several lines of evidence pointing to the role of N-linked E glycosylation in viral replication, results reported from various flaviviruses are not in complete agreement. It has been shown that the absence of E glycosylation at N154 in ZIKV results in enhanced virus binding, entry, and progeny virus infectivity in mosquito cells. However, such an effect of the E glycosylation disruption was not observed in mammalian cells [35]. Interestingly, the study using DENV showed that E glycosylation was not crucial for viral proliferation in C6/36 cell line, while E glycosylation at N67, not 153, was critical for virion release in mammalian cells [28]. In addition to mammals and insects, avians are also the amplifying and reservoir hosts for several flaviviruses, such as WNV and DTMUV. Nevertheless, the role of flavivirus E glycosylation on viral replication has not been extensively studied in avian cells compared to that in mosquito and mammalian cell lines [22]. Previously, it has been shown that the deletion of the glycan at the N154 of WNV E protein resulted in little effect on viral replication in avian cells [33]. In stark contrast, we show in the present study that the glycosylation of DTMUVE plays a pivotal role in efficient virus multiplication in both avian and mosquito cells.

Flaviviruses achieve their viral entry through receptor-mediated endocytosis [16]. C-type lectins are the major attachment factors for flaviviruses, and bind the carbohydrates on flavivirus E protein [42,43]. Two cell-surface C-type lectins, DC-SIGN, and DC-SIGNR, were implicated in flavivirus entry to the mammalian cells [22]. Accumulating evidence has demonstrated the contribution of E protein glycosylation in this process in many flaviviruses and host cells. For example, E glycosylation at N154 has been shown to facilitate ZIKV infection of Raji cells expressing exogenous DC-SIGN or DC-SIGNR [31]. Moreover, Davis et al. reported that the DC-SIGNR mediation of WNV infection is more efficient than that of DC-SIGN, particularly in human cells. This was mainly due to the glycosylation at N154 of the E protein [44]. In mosquitoes, galactose-specific C-type lectin-1 (mosGCTL-1), a soluble C-type lectin in *A. aegypti*, is associated with the glycans of WNV E proteins to facilitate the virion attachment via binding to mosquito protein-tyrosine phosphatase 1 (mosPTP-1) located on the cell surface [45,46]. Even though the role of lectin in flavivirus entry in avian cells has not been fully elucidated, the expression of C-type lectin molecules DC-SIGN was found to mediate efficient infection of infectious bronchitis virus, an avian coronavirus, even in the absence of avian-specific receptor [47]. In this study, we demonstrated that the disruption of the DTMUV E protein’s glycosylation site could significantly reduce the binding affinity of viruses to the cell surface in avian and mosquito cells. It is, however, not yet known whether avian or mosquito lectins bind to the N154 glycosylation of DTMUV E to facilitate virus entry.

Besides its role in viral entry, flavivirus E glycosylation can also influence viral particle infectivity [22]. For example, deletion of the N154 glycosylation on ZIKV E protein resulted in increased virion infectivity in C6/36 cells [35]. However, disruption of N-glycosylation of the ZIKV E protein at N154 significantly impaired secretion of the E protein, virion production, and infectivity in mammalian cells [48]. We showed here that the deletion of DTMUV E protein glycosylation at N154 decreased virion release and infectivity in both avian and mosquito cells (Figure 4). The reduction of virion release may be due to impaired virion secretion along the cellular ER-Golgi secretory pathway in the absence of N154-glycan on E protein [22]. It is also important to note that loss of N-linked glycan on E protein may also cause incorrect E folding or decrease the stabilization of the antiparallel E dimer, leading to impaired virion infectivity [22,49].

Although several studies have reported that E protein glycosylation contributes to pathogenesis in some flaviviruses [22], the function of DTMUV E protein glycosylation in neurovirulence has not been fully explored. A recent study based on reverse genetics to abolish the E protein glycosylation of ZIKV strain H/PF/2013 showed that nonglycosylated ZIKV mutants are attenuated in IFNAR1-deficient mice via subcutaneous injection, compared to the wild-type virus, giving rise to lower viral loads in brain and serum [31]. Interestingly, two research groups have independently reported that removing E protein glycosylation does not significantly affect ZIKV neurovirulence in mice via intracranial inoculation [31,35]. Our results in the current study provide evidence that deletion of the N154 glycosylation site in the DTMUV E protein not only impaired viral growth in the sera, brain, spleen, and liver in ducks, but also showed no obvious pathogenicity in ducks via an intracerebral injection route (Figure 6). These findings clearly indicate that E glycosylation is a critical molecular determinant of virulence in DTMUV. Notably, eliminating the E glycosylation significantly decreased DTMUV-induced inflammatory cytokines expression, including TNFα, IL-1β, and IL-6, in both duck primary glial cells *in vitro* and the inoculated brains *in vivo* (Figures 5 and 8). The decreased levels of inflammatory cytokines in the nonglycosylated DTMUV-inoculated ducks may reduce brain damage and neurovirulence (Figure 7). It is worth noting that, despite no mortality observed in the nonglycosylated DTMUV-inoculated ducks, many ducks still exhibited mild neurological signs characterized by walking reluctantly or discordantly, indicating that the N154 glycosylation site might not be the solely molecular determinant of virulence for DTMUV. In line with this speculation, Sun et al. have recently reported that the T367K mutation of the E protein also strongly impacts the pathogenicity of DTMUV *in vivo* [50].

In summary, our results in this study underscore the critical role of glycosylation of the E protein of DTMUV in viral pathogenesis, especially neurovirulence, in ducks. The fact that DTMUV lacking the E protein glycosylation is attenuated *in vivo* might represent a promising strategy for future DTMUV vaccine development.

## Supplementary Material

Supplemental Material
