# Supplementary material for: Glycosylation on envelope glycoprotein of duck Tembusu virus affects virus replication in vitro and contributes to the neurovirulence and pathogenicity in vivo
Source: Virulence. 2021 Sep 10;12(1):2400–14. doi: 10.1080/21505594.2021.1974329 (PMC8437475; doi:10.1080/21505594.2021.1974329)
Supplement: Supplemental Material [file KVIR_A_1974329_SM6745.docx]

**Supplementary Material**

**Table S1.** Primers used in this study.

| **Primer** | **Sequence (5’-3’)** | **Purpose** |
| --- | --- | --- |
| E-N154Q-F^a^ | CGCCTACCACcAgTATTCAGCCC | Glycosylation mutation |
| E-N154I-F | CGCCTACCACAtaTATTCAGCCC | Glycosylation mutation |
| E-N154Q/I-R^b^ | CTCGCTTCCGTGGAACCA | Glycosylation mutation |
| DTMUV-5’GSP | ACAAAGTCTCGGTTCTGCATCCCC | Verify 5’-terminal sequences |
| DTMUV-3’GSP | GACAGATTCGCCACAGCACTGCAT | Verify 3’-terminal sequences |
| DTMUV-1F | ATGTCTAACAAAAAACCAGGAAGACCC | Genome sequencing |
| DTMUV-1R | GTTGCCTTGGGATTATGAGCCTC | Genome sequencing |
| DTMUV-2F | GATGCAGAACCGAGACTTTGTTG | Genome sequencing |
| DTMUV-2R | GATCTGTCCTTTCCCACTCCCTA | Genome sequencing |
| DTMUV-3F | GAAGAATCCTACCGACACTGGGC | Genome sequencing |
| DTMUV-3R | CACTGCGGTGTCACAATCAGTTG | Genome sequencing |
| DTMUV-4F | CGTTGATGGAGAACTCATGTACG | Genome sequencing |
| DTMUV-4R | TCCTCCTCAGTAGGAATCCAATC | Genome sequencing |
| DTMUV-5F | CAGGAGATTCTGAGAAGGCGCAT | Genome sequencing |
| DTMUV-5R | CAGGAAGTATGGGTTGTATGAGG | Genome sequencing |
| DTMUV-6F | GTGGCAGTGGTAACAGGCTTCTC | Genome sequencing |
| DTMUV-6R | CTGTCAAAGTCGCGTGACACATC | Genome sequencing |
| DTMUV-7F | CATCCAGGTTCAGGAAAGACCAG | Genome sequencing |
| DTMUV-7R | CCCCATCAATTGCGTCAACCTTG | Genome sequencing |
| DTMUV-8F | ACCAGTGATTATTGAGGATGGAG | Genome sequencing |
| DTMUV-8R | CTGGGATCAAAACAATCATCAGC | Genome sequencing |
| DTMUV-9F | GAACTGCCTGAGACGCTTGAAAC | Genome sequencing |
| DTMUV-9R | GTGGTTGCATTCCAAATACGACC | Genome sequencing |
| DTMUV-10F | GAGAACTGCTGCAGGTGTGATG | Genome sequencing |
| DTMUV-10R | GCTCACCCAGTACATCTCATGTG | Genome sequencing |
| DTMUV-11F | GTGACATAGGTGAAGCTTCACCC | Genome sequencing |
| DTMUV-11R | GGGCTTCGAACTCTAGGAATCTG | Genome sequencing |
| DTMUV-12F | GTTTGAGGAGCAACACAAATGGG | Genome sequencing |
| DTMUV-12R | GGTCTCGACATGGAACCACAATC | Genome sequencing |
| DTMUV-13F | GATGTATGGAAGGGGAGGAGTTG | Genome sequencing |
| DTMUV-13R | TTACAAGACACCTTCACTCCAGC | Genome sequencing |
| DTMUV-qF | ACCATGGACAGGGTCATCAG | Quantification of viral RNA |
| DTMUV-qR | GGAGGGCTCCTTCTTGTGAT | Quantification of viral RNA |
| TNF-α-qF | CATTTGGAAGCAGCGTTTGG | Quantification of TNF-α mRNA |
| TNF-α-qR | GGTTGTGGGACAGGGTAGGG | Quantification of TNF-α mRNA |
| IL-1β-qF | AGCAGCCTCAGCGAAGAGA | Quantification of IL-1β mRNA |
| IL-1β-qR | GTCCACTGTGGTGTGCTCAGA | Quantification of IL-1β mRNA |
| IL-6-qF | CGTGTGCGAGAACAGCATG | Quantification of IL-6 mRNA |
| IL-6-qR | GTCTCGGAGGATGAGGTG | Quantification of IL-6 mRNA |
| IL-8-qF | CCTGGTAAGGATGGGAAACG | Quantification of IL-8 mRNA |
| IL-8-qR | CGTCAGCTTCACATCTTGAATAGA | Quantification of IL-8 mRNA |
| IL-12-qF | CAGCTAATAGCCATGAAGTT | Quantification of IL-12 mRNA |
| IL-12-qR | GTAGTTCTTTGCTTCACATT | Quantification of IL-12 mRNA |
| CCL5-qF | CCCAGACGAAGGAGAAACCT | Quantification of IL- CCL5 mRNA |
| CCL5-qR | AGATGGTTGTGTCAGCTCCA | Quantification of IL- CCL5 mRNA |
| GAPDH-qF | CAAGGCTGAGAATGGGAAACTT | Quantification of GAPDH mRNA |
| GAPDH-qR | GCATCTGCCCACTTGATGTT | Quantification of GAPDH mRNA |

^a^Forward primer; ^b^Reverse primer.
